# Percolated Si:SiO_2_ Nanocomposites: Oven- vs. Millisecond Laser-Induced Crystallization of SiO_x_ Thin Films

**DOI:** 10.3390/nano8070525

**Published:** 2018-07-13

**Authors:** Erik Schumann, René Hübner, Jörg Grenzer, Sibylle Gemming, Matthias Krause

**Affiliations:** 1 Helmholtz-Zentrum Dresden-Rossendorf (HZDR), Institute of Ion Beam Physics and Materials Research, Bautzner Landstraße 400, 01328 Dresden, Germany; e.schumann@hzdr.de (E.S.); r.huebner@hzdr.de (R.H.); j.grenzer@hzdr.de (J.G.); s.gemming@hzdr.de (S.G.); 2 Institute of Physics, Technische Universität Chemnitz, 09107 Chemnitz, Germany

**Keywords:** silicon, nanostructures, percolated networks, nanocomposites, thin films, laser processing, phase separation, liquid phase crystallization

## Abstract

Three-dimensional nanocomposite networks consisting of percolated Si nanowires in a SiO${}_{2}$ matrix, Si:SiO${}_{2}$, were studied. The structures were obtained by reactive ion beam sputter deposition of ${\mathrm{SiO}}_{x}$ (x ≈ 0.6) thin films at 450 ${}^{\circ }$C and subsequent crystallization using conventional oven, as well as millisecond line focus laser treatment. Rutherford backscattering spectrometry, Raman spectroscopy, X-ray diffraction, cross-sectional and energy-filtered transmission electron microscopy were applied for sample characterization. While oven treatment resulted in a mean Si wire diameter of 10 nm and a crystallinity of 72% within the Si volume, almost single-domain Si structures of 30 nm in diameter and almost free of amorphous Si were obtained by millisecond laser application. The structural differences are attributed to the different crystallization processes: conventional oven tempering proceeds via solid state and millisecond laser application via liquid phase crystallization of Si. The five orders of magnitude larger diffusion constant in the liquid phase is responsible for the three-times larger Si nanostructure diameter. In conclusion, laser treatment offers not only significantly shorter process times, but moreover, a superior structural order of nano-Si compared to conventional heating.

## 1. Introduction

Since the introduction of the first transistor [1,2,3], silicon-based technology has determined the technological progress in the world significantly, and it has changed the way of life of our society in many areas. Despite great progress and expectations raised by other materials, silicon is still the material of choice for the further development of key technologies like nanoelectronics, photovoltaics, light emitting or energy storage [4,5]. Silicon is the second most abundant element in the Earth’s crust and, hence, has the potential to promote a sustainable technology progress.

Silicon nanostructures can be based on spherical/dot-like or cylindrical/wire-like geometries. Nanodots are usually supported by an insulating silicon dioxide matrix, which constrains electrical conduction [6]. Wire-like nanostructures, on the other hand, are usually not supported by an additional matrix [4]. However, when they are in direct contact with air, oxidation leads to a few nm-thin native oxide layer, and additional near-surface defects are formed, lowering the electrical performance of these structures.

An alternative approach to obtain nm-sized Si structures is the formation of a nanocomposite of percolated Si in a SiO${}_{2}$ matrix. As first proposed by kinetic Monte Carlo (kMC) simulations [7,8], such structures should be formed if a Si-rich oxide, ${\mathrm{SiO}}_{x}$, undergoes a phase separation into Si and SiO${}_{2}$ on the nanometer scale, following Equation (1):

(1) $${\mathrm{SiO}}_{x}\to \frac{x}{2}{\mathrm{SiO}}_{2}+\left(1-\frac{x}{2}\right)\mathrm{Si}.$$

According to the kMC simulations, the Si nanostructures would be interconnected and are expected to exhibit electrical conductivity in a regime of x≤1.1. The need for alternative doping mechanisms, such as interface effects, for the Si nanocrystal-SiO${}_{2}$ system was shown by density-functional theory calculations, predicting ineffective yields for classical phosphorous doping [9,10]. Furthermore, these structures should exhibit the advantage of a tunable band gap due to quantum confinement, as predicted by atomistic pseudopotential computations [11], by density functional theory calculations [12,13,14,15,16] or by molecular dynamics [17]. Müller et al. [7] experimentally validated percolated Si:SiO${}_{2}$ nanocomposites by forming a Si-rich oxide via implantation of Si ions into a thin SiO${}_{2}$ layer. Friedrich et al. [18] and Ilday et al. [19] used physical vapor deposition to obtain ${\mathrm{SiO}}_{x\approx 1}$. Chemical vapor deposition was used by Gundogdu et al. [20] for SiO${}_{0.8}$ layer fabrication.

Up to now, the as-prepared silicon oxide thin films were processed in an oven, via rapid thermal processing [18,19] at temperatures of 950 ${}^{\circ }C$–1150 ${}^{\circ }C$ for 0.5 min to 60 min or by a point-focus laser beam. After the thermal treatment, the predicted interconnectivity was found [18,19], and even quantum confinement was observed [19]. However, when working with temperature-sensitive substrates or layer structures, only a low-temperature or a very short high-temperature process step can be used for thermal processing in order to keep both unaffected. Such temperature restrictions exist for example in thin film solar cells by the choice of the substrate (float glass, foil). Many industrial applications require processing large areas, in a short time with limited heat exposure of the substrate material. So far, these demands have not been fulfilled for percolated Si:SiO${}_{2}$ nanocomposites. Moreover, primarily the existence of percolated Si in SiO${}_{2}$ was proven, while the investigation of structural properties like compositional homogeneity, degree of Si crystallinity, Si grain size and strain remained open.

A smart technological solution to tackle the above-mentioned requirements is line-focus laser processing. Thereby, a mono-energetic beam of coherent light is scanned across the material. This induces heat for a very short time by absorption of light in a defined depth of a thin film, leaving the substrate comparably cold. Beyond depth selectivity and substrate protection, a line-focused diode laser beam enables a homogeneous treatment of a full sample. Line-focused laser processing has not been reported so far for the thermal treatment of ${\mathrm{SiO}}_{x}$ thin films or for the fabrication of Si nanostructures.

In this work, millisecond line-focus laser treatment was applied to SiO${}_{0.6}$ thin films, deposited by ion beam sputter deposition on fused silica. The obtained nanostructures were compared to those formed by conventional oven treatment. Rutherford backscattering spectrometry (RBS), Raman spectroscopy, X-ray diffraction (XRD) and cross-sectional transmission electron microscopy (TEM) were applied for comprehensive compositional and structural sample characterization after deposition and thermal treatment. The as-deposited films have perfect lateral and depth homogeneity. Experimental evidence for phase separation into amorphous Si (a-Si) and SiO${}_{2}$ prior to thermal treatment is presented. Apart of minor Si enrichment at the surface and the interface to the SiO${}_{2}$ substrate, the homogeneous sample composition is conserved during thermal treatments. The oven treatment resulted in Si:SiO${}_{2}$ nanocomposites whose Si volume fraction has 72% crystallinity and a mean Si diameter of about 10 nm. The Si volume fraction obtained by millisecond laser treatment was characterized by more than 90% crystallinity and almost single domain nano-crystals with about a 30 nm mean diameter. The observed structural differences are explained by different diffusion constants for the solid and liquid states of matter.

## 2. Results

**Figure 1:**
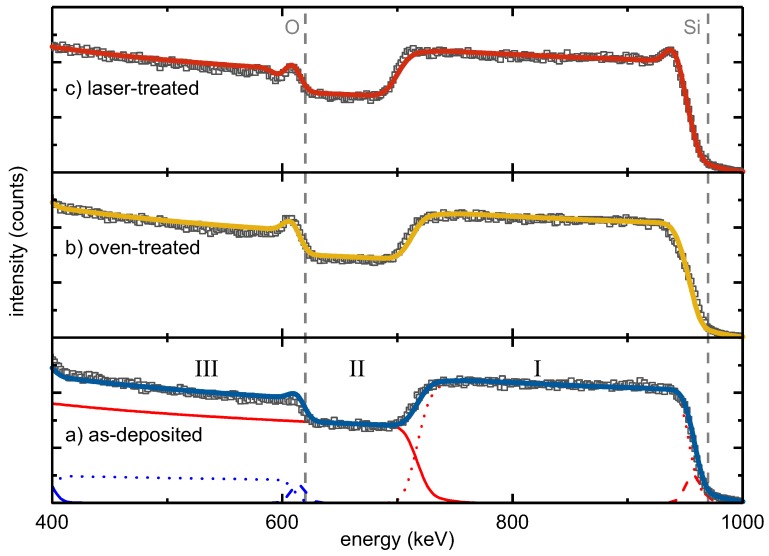
Rutherford backscattering spectrometry (RBS) spectra of SiO${}_{0.6}$ layers on SiO${}_{2}$: as-deposited (**a**), after conventional oven (**b**) and after laser treatment (**c**). The energy range between 1 keV and 1.7 keV, which shows traces of Ar and W, was omitted for clarity. Squares represent measured data and lines fitted curves. In (**a**), single-layer elemental profiles are additionally shown, i.e., red for Si and blue for O. The roman letters (I, II, III) indicate the major RBS feature types explained in the text.

Lateral and depth composition of as-deposited, oven-treated and laser-treated SiO${}_{0.6}$ were studied by RBS (Figure 1).

The energy of backscattered He ions is determined by a kinematic factor [21,22]. The heavier the target atoms, the smaller the energy loss of the scattered ion and the higher the measured backscattering energy. Moreover, the energy loss of the scattered ions is proportional to the depth of the scattering event due to electronic stopping [21,22], and thus, the larger the thickness, the smaller the measured scattering energy. The RBS intensity is proportional to the concentration of the elements in a sample. The width of an element-specific signal plateau indicates the thickness of an individual layer.

The RBS spectra of the three studied samples showed the following major features: (I) the Si signal of the SiO${}_{0.6}$ layer at backscattering energies from 970 keV to about 700 keV, (II) the Si signal from the SiO${}_{2}$ substrate starting from about 700 keV and (III) the O signal of the SiO${}_{0.6}$ from 610 keV to lower energies.

Moreover, all three spectra had a peak at the edge of the SiO${}_{0.6}$ oxygen signal in common that was attributed to a thin intentionally-deposited protective SiO${}_{2}$ top layer. Complementarily, a flattened Si edge at 970 keV was observed accounting for the reduced Si content in this surface layer compared to the SiO${}_{0.6}$ film. Correspondingly, a three-layer stack consisting of a SiO${}_{2}$ surface layer, SiO${}_{0.6}$ bulk and SiO${}_{2}$ substrate was necessary for fitting the main RBS features. Minor constituents, such as tungsten or argon, which originated from the deposition process, represented less than 0.1% of the sample composition. An overview of the obtained compositions and areal densities derived from the RBS measurements is given in Table 1. To correlate areal densities with thicknesses, TEM and spectroscopic ellipsometry (SE) measurements were conducted, the data of which are also presented in Table 1.

From the perfect agreement of the RBS data and the corresponding three-layer fit model, it follows that the elemental depth distribution within the as-deposited and the oven-treated sample was homogeneous. The RBS spectrum of the laser-treated sample showed an additional peak at the high-energy edge of the SiO${}_{0.6}$ bulk signal. This observation required the introduction of an additional Si-rich intermediate layer between the SiO${}_{2}$ surface and the SiO${}_{0.6}$ bulk, termed as Intermediate 1 (Table 1). In a similar way, a steeper slope at the low-energy edge of the SiO${}_{0.6}$ bulk Si signal was attributed to another Si-rich intermediate layer on its bottom (Table 1, Intermediate 2).

Based on the fit models, precise numbers for the atomic compositions of the three-film stacks were derived (Table 1). The SiO${}_{0.6}$ bulk layer of the as-deposited sample contained about 38.9% atomic oxygen, corresponding to SiO${}_{0.64}$ with an areal density of $2770\times 10^{15}$ cm^−2^. Compared to the as-deposited sample, the oven-treated SiO${}_{0.6}$ film did not show a significant change in bulk composition. The fitted bulk O content of 39.4% conformed to the bulk O content of the as-deposited layer within the accuracy of measurement and homogeneity of sample deposition, resulting in SiO${}_{0.65}$. The surface oxygen peak increased after oven treatment. The protective SiO${}_{2}$ surface layer apparently grew in thickness to $150\times 10^{15}$ cm^−2^ during the oven treatment, i.e., by about 50%. Furthermore, the laser-treated silicon oxide film did not show a change in the bulk composition (Table 1). Its O content of 38.4%, resulting in SiO${}_{0.63}$, was the same as for the other two films within the experimental accuracy. As mentioned before, the oxygen peak superimposed on the high-energy edge of the SiO${}_{0.6}$ bulk layer was stronger than in the as-deposited and oven-treated one. Moreover, a peak at the high-energy edge of the SiO${}_{0.6}$ Si signal was unambiguously apparent (Figure 1). At the same time, a minimum was seen next to the oxygen peak towards lower energy. Both features were described by a thin Si-rich layer, referred to as Intermediate 1, underneath the protective SiO${}_{2}$ surface layer with an areal density of $100\times 10^{15}$ cm^−2^ and a low O content of 10%. To improve the fitting of the low-energy slope of the SiO${}_{0.6}$ layer Si signal, an additional Si-rich second intermediate layer with an areal density of $50\times 10^{15}$ cm^−2^ and an oxygen content of 10% was introduced between the bulk SiO${}_{0.6}$ and the silica substrate. This analysis indicated that contrary to oven treatment, laser processing led to the formation of Si-rich interlayers at both interfaces of the SiO${}_{0.6}$ bulk layer.

**Figure 2:**
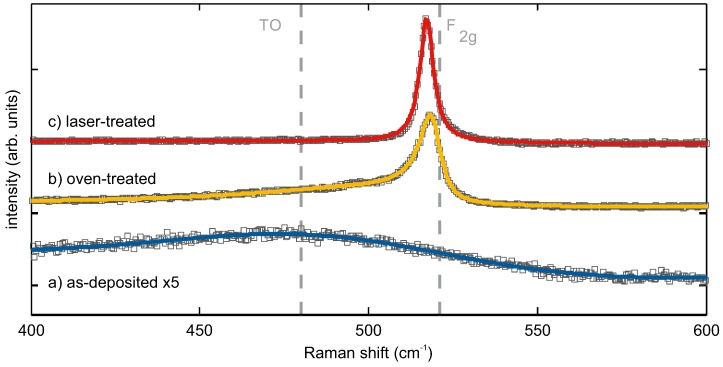
Raman spectra of SiO${}_{0.6}$ layers on SiO${}_{2}$: as-deposited (**a**), after conventional oven (**b**) and after laser treatment (**c**). Grey squares represent measured spectral data points and solid lines fitted spectra. Dashed lines indicate the expected positions of the TO-like band associated with a-Si and the F${}_{2g}$ phonon mode of c-Si.

Raman spectroscopy and X-ray diffraction are complementary methods for the analysis of phase and micro-structure, lattice strain and crystal size effects in silicon [23,24]. The Raman spectra of as-deposited, oven- and laser-treated SiO${}_{0.6}$ showed distinct differences in the range from 400 cm^−1^ to 600 cm^−1^, where the F${}_{2g}$ crystal vibration of crystalline Si (c-Si) and the broad line of a-Si were observed [25,26,27,28,29] (Figure 2).

Prior to thermal processing, the observed spectrum showed only one broad line (full width at half maximum (FWHM ≈ 100 cm^−1^)) with the peak maximum at 485 cm^−1^. It corresponded to that of a-Si [27,30] and indicated a phase separation of SiO${}_{0.6}$ into a-Si and SiO${}_{2}$ occurring already during the deposition and not only after high-temperature processing, as predicted and reported in the literature for this material system. After oven tempering, the spectrum had a sharp strong line at slightly less than 521 cm^−1^ with an extended low-energy shoulder. Fitting this Raman spectrum with one Breit–Wigner function (BWF, asymmetry factor q = −7.2) and two Gaussians gave line positions (relative integral intensities) of 519 cm^−1^ (60%), 500 cm^−1^ (6%) and 482 cm^−1^ (34%). This is the typical Raman signature of so-called micro-crystalline silicon, consisting of nanocrystalline and amorphous Si fractions [31,32]. The sharp peak at 519 cm^−1^ was attributed to the F${}_{2g}$ phonon mode of the nanocrystalline silicon fraction (c-Si). Its FWHM of 7.9 cm^−1^ was two- to three-times larger than the natural linewidth of single-crystalline silicon at room temperature reported in the literature (≈3.5 cm^−1^) [33,34] or that of a reference Si wafer sample (2.8 cm^−1^). The shoulder peak with the intensity maximum at 482 cm^−1^ represented the a-Si fraction in the sample, and the intermediate line at 500 cm^−1^ was attributed to defective Si (def-Si), i.e., wurtzite-type or near-surface Si [32,35,36,37]. Laser treatment of the SiO${}_{0.6}$ resulted in a structure, the Raman spectrum of which exhibited only one single strong peak at first glance (Figure 2). The best line fitting results (r${}^{2}$ = 0.996) were obtained assuming a BWF (FWHM = 5 cm^−1^) and a Gaussian line with Raman shifts (relative integral intensities) of 517 cm^−1^ (86%) and 470 cm^−1^ (14%).

The crystalline Si volume fraction $\rho_{c}$ is often estimated from the integral Raman intensities by Equation (2) [38]:

(2) $$\rho_{c}=\frac{I_{c-Si}+I_{def-Si}}{I_{c-Si}+I_{def-Si}+yI_{a-Si}}$$

The ratio of the crystallite size-dependent integrated Raman cross-sections, $y=\sum_{c-Si}/\sum_{a-Si}$, determines the Raman line shape of nanocrystalline Si [38,39]. Provided that a BWF line shape gave the best fitting results for the c-Si Raman profile, a minimum crystallite size of L ≥ 15 nm, and thus, the minimum *y*-value of y=0.65 was reported in the literature [38]. On the other hand, this criterion for the estimation of the crystallite size is rather weak, and thus, the mean Si crystallite size was determined here from X-ray diffraction and was then used to apply Equation (2). Another diagnostic criterion to estimate the Si crystallite size from Raman data consists in the use of the correlation between F${}_{2g}$ linewidths and crystallite sizes determined by X-ray diffraction. Comparing our FWHM of 7.9 cm^−1^ and 5.0 cm^−1^ with such a correlation from the literature [24] gave values of approximately 8 nm and > 15 nm for the oven- and laser-treated samples.

None of the three Raman spectra showed any contribution of SiO${}_{2}$, neither at first glance, nor during spectra fitting. Its absence was caused by its low Raman cross-section due to the energy gap of the order of 9 eV [40,41].

**Figure 3:**
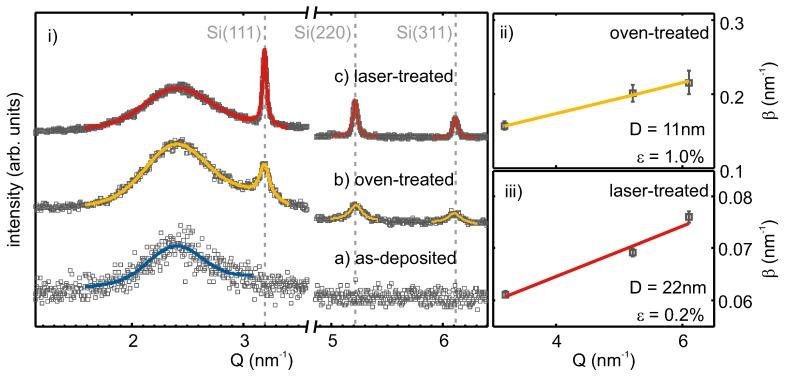
(**i**) XRD patterns of SiO${}_{0.6}$ layers on SiO${}_{2}$: as-deposited (a), after conventional oven (b) and after laser treatment (c). The broad peak at about 2.4 nm^−1^ originated from the a-SiO${}_{2}$ matrix and silica substrate. Measurement data are shown by grey squares. The solid lines represent fitted curves. (**ii**,**iii**) The determined diffraction peak width is plotted vs. its position; the linear regression was made for Williamson–Hall analysis to retrieve crystallite size D and micro-strain ϵ for the oven- (**ii**) and laser-treated (**iii**) sample.

In Figure 3i, XRD intensities of as-deposited, oven-, as well as laser-treated SiO${}_{0.6}$ are shown over the scattering vector $Q=\frac{2}{\lambda }\sin\left(\Theta \right)$, with λ being the incident beam wavelength and Θ the scattering angle. The subfigures (ii) and (iii) show Williamson–Hall (WH) plots of the oven- and laser-treated samples.

For the as-deposited film, no diffraction peaks were observed. The broad feature at ≈2.4 nm^−1^, which was also seen in the other diffraction patterns, originated from the amorphous SiO${}_{2}$ substrate and matrix. Therefore, the as-deposited film was X-ray amorphous and did not exhibit a crystalline phase. Oven treatment led to the evolution of three diffraction peaks at about 3.19 nm^−1^; 5.22 nm^−1^; 6.11 nm^−1^, which corresponded to the (111), (220) and (311) lattice planes of Si [42]. The observed linewidths were broad and showed an increase with increasing scattering vector. After laser treatment, diffraction peaks at the same positions were observed, but with higher intensity, as well as narrower linewidth. The increased intensity can be correlated with an increased scattering volume of well-ordered lattice planes (i.e., a higher density of crystallites in the layer) and the smaller linewidth results from larger crystallite sizes, compared to the oven-treated sample.

The observed peaks assigned to Si lattice planes were fitted by Lorentzians. From the obtained peak positions and the assigned Miller indices, the mean lattice spacing was calculated as 5.425(7) Å and 5.424(1) Å for the oven- and laser-treated films, respectively. These values differed at most by 0.007 Å or 0.13% from the literature value of 5.431 Å [42] for the lattice spacing of strain-free silicon. In the subsets of Figure 3, the fitted and, for instrumental resolution, corrected integral widths are plotted against the peak positions following the method by Williamson and Hall [43]. From linear regression, the Si crystallite size and the micro strain, the varying of the lattice parameter, ϵ, can be calculated following β=1/D+2ϵQ. For the oven-treated sample, a crystallite size of 11 nm and a lattice parameter variation of 1.0% were retrieved. After laser processing, larger Si crystallites of 22 nm and less lattice parameter variation (0.2%) were apparent. Together, the obtained mean lattice parameter and Williamson–Hall analysis of line broadening with the increasing scattering vector suggest a slightly compressed Si crystal lattice. This could be the result of vacancies in the Si lattice.

X-ray reflectivity studies (not shown) of as-deposited, oven- and laser-treated samples showed an increase in surface roughness after laser treatment to about 7 nm, whereas the as-deposited and oven-treated sample exhibited a surface roughness of about 3 nm.

Having determined the mean crystallite sizes by XRD, the corresponding values were used to calculate the integrated Raman cross-sections, $y=\sum_{c-Si}/\sum_{a-Si}$, for insertion into Equation (2). This gave *y*-values of 0.74 and 0.51, and crystalline Si volume fractions (CVF) of 72% and 92% for the oven- and laser-treated samples, respectively.

**Figure 4:**
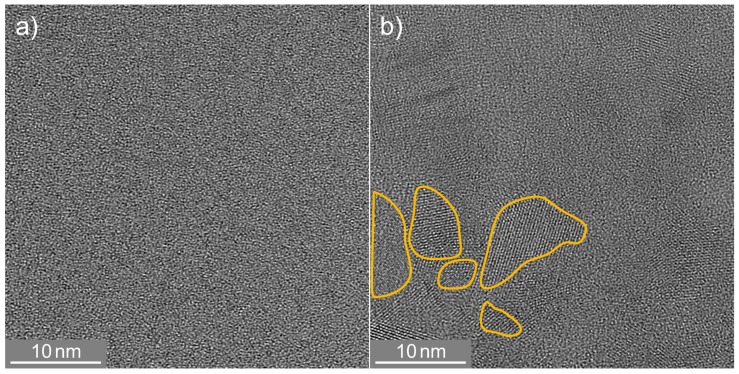
High-resolution TEM image of SiO${}_{0.6}$ layers: as-deposited (**a**) and after conventional oven treatment (**b**).

While Raman spectroscopy and X-ray diffraction allowed structural characterization on an integral scale, TEM revealed the details of the microstructure up to atomic resolution in selected, spatially-confined sample regions. High-resolution cross-sectional TEM micrographs (Figure 4a) of the as-deposited SiO${}_{0.6}$ layer did not show any hints of crystalline Si.

After oven treatment, areas with clearly noticeable lattice fringes were visible, and a selection of them is marked in Figure 4b. Lattice planes were randomly oriented, and regions with the same orientation had dimensions as large as 12 nm. The TEM lamella thickness was of the order of a few 10 nm and hence much larger than the observed crystallite size. Therefore, one should keep in mind that separated single crystallites might appear to form a connected path, due to the projection of the depth information onto a 2D image.

**Figure 5:**
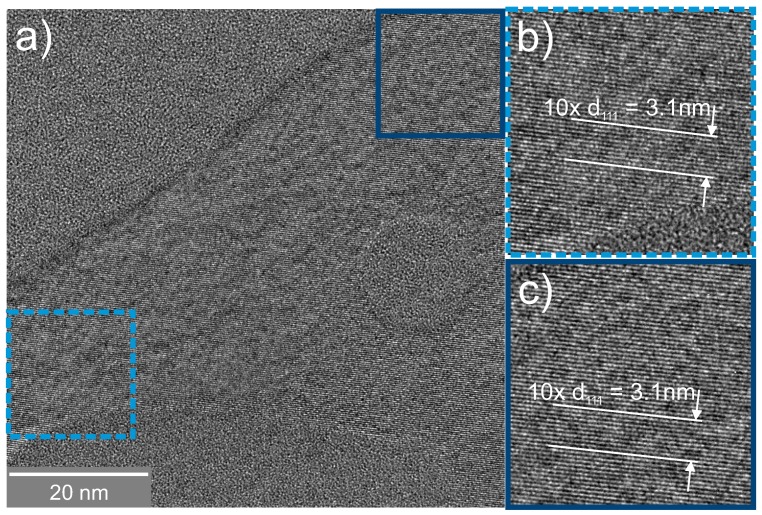
High-resolution TEM image of laser-crystallized SiO${}_{0.6}$ layers. (**a**) Si (111) lattice fringes expand across the whole field of view. The matrix surrounding the crystal is assumed to be SiO${}_{2}$. (**b**,**c**) show enlargements of diagonal opposite areas of (**a**).

In Figure 5a, an image of laser-crystallized SiO${}_{0.6}$ is shown.

Across the whole field of view, a single orientation of Si lattice planes was visible, i.e., only a single grain and no grain boundaries were seen. The two insets (b,c) depict enlargements of the diagonal corners of a crystalline Si grain. The matrix surrounding this Si crystallite was amorphous SiO${}_{2}$. An infinitesimally thin a-Si layer at the interface cannot be excluded, but was not visible due to the lack of mass contrast between SiO${}_{2}$ and Si. The distance of 10 distinct lattice planes was measured and yielded approximately 3.1 nm, resulting in a mean lattice plane spacing of 3.1 Å. This value agrees with the Si (111) lattice spacing of 3.14 Å [42].

**Figure 6:**
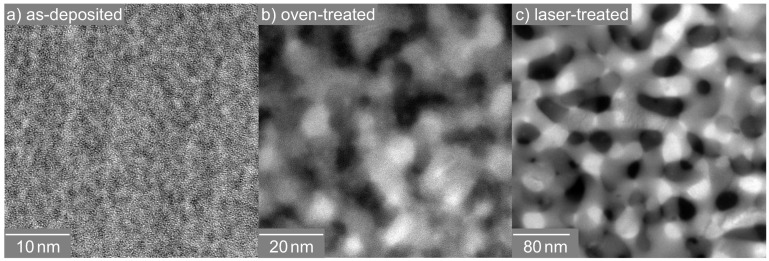
Cross-sectional Si plasmon-loss filtered TEM images (E${}_{loss}$ = 17 eV) of SiO${}_{0.6}$ layers: as-deposited (**a**), after conventional oven (**b**) and after laser treatment (**c**).

None of the techniques applied so far provided information about the spatial distribution and connection of the individual Si and SiO${}_{2}$ phases within the thin layer, i.e., about whether a nano-sized percolated structure was formed. Therefore, energy-filtered transmission electron microscopy (EFTEM) was used to resolve the spatial distribution of separated Si and SiO${}_{2}$ (Figure 6). For the Si distribution, electrons with an energy loss of 17 eV, due to the excitation of silicon plasmons, were used, while the complementary SiO${}_{2}$ distribution (not shown here) was obtained by imaging with electrons, which exhibited an energy loss of about 27 eV.

Although illuminating the particular region of the TEM lamella only for the EFTEM analysis, which was conducted as fast as possible to avoid electron-beam-induced specimen decomposition, the cross-sectional image of as-deposited SiO${}_{0.6}$ (Figure 6a) showed a distinct image contrast between Si (bright) and SiO${}_{2}$ nanoparticles (dark). The corresponding Si feature size was estimated to be about 2 nm. This result confirmed the occurrence of phase separation upon deposition. As the structure size was much smaller than the specimen thickness, by EFTEM, a conclusion about whether a projection of a number of isolated particles or a percolated structure was present cannot be drawn. After oven processing (Figure 6b), the Si plasmon-loss filtered TEM image revealed a coarsened microstructure with a feature size of about 10 nm. Again, it was not definite whether the observed Si structures were fully percolated. The laser-processed film (Figure 6c) consisted of, compared to oven-treated structures, a similar Si morphology with a much broader size. The measured mean bar width yielded 30 nm. Since these structure sizes were comparable to the TEM specimen thickness, the formation of a percolated Si network was unambiguously shown in this case. Friedrich et al. [18] and Liedke et al. [8] reported percolated Si structures with a mean feature size of 3 nm for Si:SiO${}_{2}$ systems with a lower Si volume concentration. Based on the EFTEM analysis (Figure 6), the higher Si volume fraction compared to Friedrich et al. [18], Liedke et al. [8] and percolation theory, it was deduced that three-dimensional percolated Si structures were present in all SiO${}_{0.6}$ thin films of our study.

## 3. Discussion

In the previous section, the formation of percolated a-Si:SiO${}_{2}$ networks during ion beam sputter deposition at 450 ${}^{\circ }C$ is shown. The occurrence of phase separation during deposition is independently shown by Raman and EFTEM, in contrast to previous studies of this material system. By thermal treatment, crystallization of a-Si and coarsening of the initial network occurred. In the following, (i) the structural and compositional homogeneity of the samples, (ii) the structural properties of the three layer types, (iii) the origin of the different structure sizes and (iv) the processes leading to the formation of Si-rich interface layers are discussed.

### 3.1. Structural and Compositional Homogeneity

As-deposited, oven- and laser-treated SiO${}_{x}$ films of this study were shown to have the same bulk composition of SiO${}_{0.64\pm 0.06}$.

The lateral composition was checked at five 1 mm${}^{2}$ large areas of the initial 20 mm × 20 mm as-deposited sample. It was found to be the same within experimental accuracy. A measurement uncertainty of 2% for the total areal density and the areal density of the elemental Si results in an uncertainty for *x* of 0.06. Across the whole as-deposited sample, a thickness variation of 10% was observed by RBS.

Comparing the depth compositions, the total areal densities per element of all three samples retrieved by RBS agree very well within the experimental accuracy and sample homogeneity. Layer thicknesses obtained by TEM and SE agree within experimental accuracy (5%) and sample thickness homogeneity. Total layer thickness differs between as-deposited and thermally-treated films by <5%. Hence, no loss in material occurred after thermal treatment. The formation of new layers and growth of oxide layers occurred at the expense of the as-deposited SiO${}_{0.6}$ layer.

All presented measurements were conducted randomly on the 5 mm × 5 mm cut-out of the initial sample. No dependency of the measurement results on sample position was found. Hence, observed structures of the as-deposited, oven- and laser-treated SiO${}_{0.6}$ layers are homogeneous across the samples.

It can be stated that deposition, as well as thermal treatment are suitable for large area, homogeneous formation of Si:SiO${}_{2}$ nanostructures.

### 3.2. Comparison of Structural Properties

A comparison of the structure properties and treatment parameters is shown in Table 2.

Oven treatment of the as-deposited, phase-separated Si:SiO${}_{2}$ structure leads to a coarsening of the as-deposited morphology and to crystallization of the main fraction of the initially amorphous Si. The result is a coexistence of crystalline and a minor fraction of a-Si. The crystallites have a random orientation and a mean grain size of 11 nm. XRD analysis yields a lattice parameter variation of about 1%, with a tendency to smaller values of the lattice parameter.

Silicon nanostructures obtained by laser treatment of SiO${}_{0.6}$ exhibit a three-times larger structure size than those obtained by oven processing. Its crystalline volume fraction is much higher, i.e., almost no amorphous Si phase was observed. Obtained Si crystallites exhibit a mean grain size of 22 nm and are extended for up to 100 nm. The XRD lattice parameter is reduced by 0.2% compared to a Si powder reference. No signs of crystallized SiO${}_{2}$ were found for both thermal processing methods.

A decrease of the Raman frequency of the peak associated with crystalline Si is observed for both oven- (2 cm^−1^) and laser- (4 cm^−1^) treated samples. A decrease in the resonance frequency of the collective lattice motion can be caused by phonon confinement or by an increased mean lattice spacing compared to the reference due to the presence of strain [24,44,45,46,47,48] or by an increased temperature [29]. The grain sizes derived by EFTEM and complementarily by XRD analysis yield structure sizes too large for phonon confinement, since for this effect, a typical size below 10 nm has to be reached [4]. Sample heating during Raman measurement was excluded, since no dependence of the applied laser power on the Raman signal was found.

XRD measurements shown above yield a deviation of the mean lattice spacing compared to bulk silicon. The observed lattice parameter were at most 0.13% smaller than for the bulk Si literature value, suggesting a compressed silicon lattice. Likewise, Williamson–Hall analysis yielded a lattice parameter variation of the order of 1.0% for oven- and 0.2% for laser-treated films. On the other hand, a down-shift of the Raman peak of 2 cm^−1^ to 4 cm^−1^ would suggest an expanded lattice, due to a tensile stress of 1.7 GPa to 3.6 GPa [49,50]. Such stress would lead to a lattice expansion of 0.39–1.38%. Hence, Raman results seem to contradict the XRD results. However, due to the measurement geometries applied, XRD probes the out-of-plane Si lattice distances, whereas Raman in the applied 180${}^{\circ }$ scattering geometry is sensitive to the in-plane Si geometries. During heat treatment, relaxation of stresses at Si-SiO${}_{2}$ interfaces takes place, which is reduced and eventually inhibited during cooling. The thermal expansion coefficient for the SiO${}_{2}$ substrate (α≈ $0.5\times 10^{-6}$ K^−1^ [51]) is much smaller than the thermal expansion coefficient for Si (α≈ $2.6\times 10^{-6}$ K^−1^ [52,53]). It follows that the contraction of the Si phase in the thin film is inhibited mainly by the relatively lower contraction of the SiO${}_{2}$ substrate. This in-plane tensile stress results in an in-plane expansion of the lattice. To compensate the in-plane expansion, a compression of the out-of-plane Si lattice components follows.

### 3.3. Origin of Different Structure Sizes

An objective is to gain insight into the formation processes, leading to Si:SiO${}_{2}$ nanocomposites. In general, during the thermal treatments, two processes must take place: (a) growth of the structure size of the as-deposited nano-network of the Si:SiO${}_{2}$ nanocomposite and (b) crystallization of the Si phase. The initial point is a mixture of as-deposited a-Si and SiO${}_{2}$, as was observed by Raman, as well as by EFTEM measurements. Certainly, a fully phase-separated material with sharp interfaces cannot be assumed after deposition. Most likely, transition regions between the two phases with an amorphous ${\mathrm{SiO}}_{x}$ exist, where thermal treatment leads to an enhanced phase separation.

Generally, during the growth of the as-deposited Si:SiO${}_{2}$ nano-composite, diffusion of Si- and/or O- atoms has to take place in the silicon and/or silica phase. Bulk diffusivity of Si-and O atoms in the silica phase is rather low (Si: $1\times 10^{-19}$ cm${}^{2}$ s^−1^ [54,55]; O: $1\times 10^{-16}$ cm${}^{2}$ s^−1^ [55]) for the temperature applied during oven treatment. Similarly, Si self-diffusion by self-interstitial or vacancy transport is limited to $1\times 10^{-17}$ cm${}^{2}$ s^−1^ [55] at 950 ${}^{\circ }C$. The diffusion of O atoms in silicon by interstitial transport, on the other hand, reaches diffusion constants of $1\times 10^{-11}$ cm${}^{2}$ s^−1^ at 950 ${}^{\circ }C$, which is five orders of magnitude larger than any other regarded diffusion possibilities. In addition to bulk diffusion, Si and O atoms can diffuse along grain boundaries, which is about 4–8 orders of magnitude faster [56]. Approaching the melting point of a material, the diffusivity of grain boundary diffusion converges to the bulk diffusivity [55]. Hence, the relevant transport for the coarsening of the Si:SiO${}_{2}$ nanocomposite is the one of O atoms in the silicon matrix by interstitial transport and the transport of Si and O atoms along grain boundaries.

Observed in this work are a considerable smaller structure size and lower crystalline volume fraction after oven treatment compared to laser processing, while the treatment time is seven orders of magnitude longer. The temperature for oven treatment is 950 ${}^{\circ }C$, whereas the temperature achieved during laser processing cannot be estimated easily, since physical properties like thermal conductivity and heat capacity are not known for the nanocomposite material investigated. Furthermore, the absorption coefficient might change during laser treatment. A viable, legitimate estimation of the laser-induced temperature by analytical [57] or numerical [58] methods would therefore require rigorous and careful considerations, beyond the scope of this work. However, the energy input for laser treatment can be estimated as follows. During laser treatment, an energy of 98 J cm^−2^ is applied to the sample, taking the optical laser power and dwell time into account. When regarding the absorption of the laser wavelength by the SiO${}_{0.6}$ layer, 21 J cm^−2^ are absorbed by the material. In contrast, an energy of 210 J cm^−2^ is needed to heat the substrate and the thin film, about 0.1 J cm^−2^ is due to the SiO${}_{0.6}$ film. It is straightforward that a more evolved nanostructure requires more energy to be formed, i.e., it requires either longer processing times or higher temperatures. Therefore, since the processing time during laser treatment is much shorter, a higher temperature must have been achieved.

When considering a solid state process, the diffusion constant of O atoms in the Si-phase rises to $4\times 10^{-9}$ cm${}^{2}$ s^−1^, just below the melting point of silicon [59]. This represents an increase by three orders of magnitude, not sufficient to explain the observed coarsening of the laser-treated Si:SiO${}_{2}$ nanocomposite structure. A further rise in temperature, resulting in a process occurring in the liquid state, leads to a sudden increase of the diffusion of O atoms in the Si-phase to $3\times 10^{-4}$ cm${}^{2}$ s^−1^ [59], i.e., by five orders of magnitude. A similar rise upon melting is expected for the diffusion along grain boundaries. This now fully conforms with the experimental findings. We therefore conclude that during laser treatment, growth and enhanced phase separation occurs in the liquid state of silicon. The temperature window can now be assumed to range from the melting temperature of a-Si to that of SiO${}_{2}$, i.e., from 1200 ${}^{\circ }C$ [60,61] to 1705 ${}^{\circ }C$ [62,63], since a breakdown of the general, percolated morphology would be expected for a system consisting of two liquids.

Crystallization during oven treatment is regarded as a classical nucleation and growth process. The observation of many randomly-oriented nanocrystals supports the mechanism of nucleation at various places and subsequent growth by heat treatment. During laser processing, vast Si crystallites and an almost full crystallization of the Si-phase were obtained. A thermal process via liquid state leads to a contraction of the Si-phase, since Si has a higher density in the liquid ( 2.52 g/cm${}^{3}$ [64,65,66]) than in the solid (crystalline Si: 2.33 g/cm${}^{3}$; amorphous Si: 2.29 g/cm${}^{3}$) phase. SiO${}_{2}$, on the other hand, expands little during heating [51]. During solidification and cooling, the Si-phase expands, while being confined in the low-expansion material SiO${}_{2}$. Since the density of crystalline Si is higher compared to the amorphous state, full crystallization is favorable.

The vast Si grains can be explained, in contrast to nucleation and growth, by a process similar to explosive crystallization [67,68]. There, latent heat, released during solidification and crystallization, causes melting of adjacent silicon, resulting in a self-propagating liquid region traveling through the as-deposited amorphous Si:SiO${}_{2}$ layer. Now, the scanning laser controls the velocity of this process. The crystallization occurs along the moving liquid-solid interface with already crystallized Si acting as the seed for the growing crystal grains.

Ideally, the crystallite size would have shown a sudden increase from that of the solid-state to liquid phase crystallization after a threshold power density was achieved. Such a threshold could not be detected in the present study. On the other hand, laser-induced liquid phase crystallization of silicon on glass was demonstrated for 808 nm laser irradiation at lower power densities than that used here [69,70].

As an alternative mechanism for laser treatment-induced crystallization, enhanced atomic diffusivity due to local electronic excitations shall be briefly discussed. It was reported for the crystallization of amorphous TiO${}_{2}$ and TiON, respectively, upon UV laser irradiation [71,72]. This process is based on randomly-distributed local atomic excitations and resulted in statistically-distributed TiO${}_{2}$ nanocrystals in an amorphous matrix as observed in the work of Teodorescu et al. [72]. Hence, even though the conditions for electronic excitations of a-Si by a laser energy of 1.53 eV (808 nm), i.e., a direct bandgap of approximately 1.7 eV, are nearly fulfilled, the resulting phase and microstructure of the laser-treated Si:SiO${}_{2}$ nanocomposite precludes such a crystallization pathway.

In conclusion, both, almost full crystallization and vast Si grains, support the assumption of a process occurring in the liquid state.

### 3.4. Formation of Interface Layers

During thermal treatment, surface and interface layers grow or even evolve. After oven treatment, the initially-deposited SiO${}_{2}$ cover layer more than doubles in thickness. Following laser treatment, the cover layer also grows in size, and additional silicon-rich interface layers form.

The growth of the cover layer can be explained by further oxidation during thermal treatment. Oven processing was conducted in Ar atmosphere, but residual and effused O${}_{2}$ from the as-deposited layer, as well as O atoms from interstitial positions in the Si-phase can still cause oxide formation. Laser treatment was done at normal atmospheric conditions.

Additional Si-rich interface layers observed for laser-treated samples can be explained by the presence of the adjacent silicon oxide surface layer, as well as the silica substrate. At first, SiO${}_{2}$ grows at the interface with these layers, favored by a lower surface energy of the planar layers compared to SiO${}_{2}$ nanostructures, leading to an enhanced wetting and preferred growth of the SiO${}_{2}$ at the substrate and surface oxide layer. For a certain distance to these interface layers, due to the diffusion length of O atoms during thermal treatment, a volume depleted of oxygen forms. Hence, the formation of a silicon-enriched intermediate layer is caused. In principle, this process should also occur for oven-treated samples. Since diffusion during oven treatment is low compared to laser processing, this effect was not resolved for the treatment process used in this study.

## 4. Materials and Methods

### 4.1. Sample Deposition and Processing

SiO${}_{0.6}$ films of 500 nm in thickness were prepared by reactive ion beam sputter deposition [73,74] on 20 mm × 20 mm fused silica substrates. An argon ion beam of 1 keV energy and 35 mA current from a 3 cm Kaufman-type ion source (Ion Tech inc., Fort Collins, CO, USA) was applied to sputter a 6 silicon target (99.999%). The ion-source-to-target distance was 18 cm, and the target was tilted by an angle of 22 with respect to the ion beam. The sample was positioned 18 cm away from the target and heated to 450 ${}^{\circ }C$ by a boron nitride heater. The base pressure was $2\times 10^{-5}$ Pa, and the working pressure was $8\times 10^{-3}$ Pa. By injection of 1.5 sccm oxygen into the sputtering chamber, that raised the working pressure by $1\times 10^{-3}$ Pa, and a stoichiometry of SiO${}_{0.6}$ was obtained. For protection against post-deposition oxidation, a 15 nm SiO${}_{2}$ top layer was deposited. After cutting the as-deposited sample, a comparative post-deposition treatment was performed by two approaches. Three individual isothermal oven treatments of 90 min at 950 ${}^{\circ }C$ with intermediate cooling, all under Ar atmosphere, were applied to one part of the sample. The other part was laser-treated at ambient conditions using a 808 nm radiation of an Activation Line 450 diode laser (LIMO, Dortmund, Germany) emitting a Gaussian-shaped line focus of 11 mm × 0.1 mm and a maximum power density of 11.7 kW cm${}^{2}$. A dwell time, assumed to be the width at $\frac{1}{e^{2}}$ of the maximum power density passing by the feed rate of the supposed ideal laser beam, of 13 ms was used.

### 4.2. Characterization

Elemental composition and depth distribution of the SiO${}_{0.6}$ thin films before and after post-deposition treatment were analyzed by RBS (Transformatoren- und Röntgenwerk, Dresden, Germany) using helium ions with an energy of 1.7 MeV from a Van de Graaff accelerator. The obtained spectra were fitted with the help of the SIMNRA [75] software (version 6.06, Max-Planck Institut für Plasmaphysik, Garching, Germany).

The thin film thicknesses were determined by rotating compensator spectroscopic ellipsometry (SE; M-2000FI from J. A. Woolam Co., Lincoln, NE, USA). The ellipsometric angles Psi and Delta were recorded in the range from 210 nm–1680 nm at a fixed angle position of 75. Thickness was obtained by fitting a layer stack model based on reference data for the refractive indices to the measured data. The resulting simulated stack for the as-deposited sample consists of three parts, a fused silica substrate, a bulk layer, which was described by a Bruggeman effective medium approximation (EMA) with different amounts of silicon dioxide [76] and amorphous [40] silicon and a silicon dioxide top layer. For the thermally-treated samples, Bruggeman EMAs were expanded by adding contributions of crystalline [76] silicon. To validate the obtained thicknesses, selected samples were cross-checked with profilometry using a DEKTAK 8000 (Veeco, Mannheim, Germany) equipped with a 12.5 μm stylus, and punctually by cross-sectional TEM.

Raman spectra were measured with a micro-Raman LabramHR spectrometer (Horiba, Bensheim, Germany). For excitation, the beam of a frequency-doubled Nd:YAG laser with a wavelength of 532 nm was focused on the samples using a long-working distance objective with 100-fold magnification. The laser power density on the sample was minimized to 3 kW cm^−2^ (0.1 mW laser power) in order to avoid any thermally- or photo-induced transformation of Si. The collected Raman-scattered light was dispersed by an 1800 mm^−1^ holographic grating and recorded with a liquid nitrogen cooled CCD detector.

X-ray diffraction was performed with an Empyrean Θ-Θ 4-circle diffractometer (Panalytical, Almelo, Netherlands) using the 0.154 nm Cu-Kα line. A parallel beam was established using a parabolic X-ray mirror and a 2 mm fixed mask. An anti-scatter slit of 1.4 mm and a 1/8 ${}^{\circ }$ fixed divergence slit were used to form the beam incident on the sample at 1°. For the diffracted beam, Soller slits ( 0.04 rad) and a parallel plate collimator with an opening of 0.27° were used. A proportional Xenon point detector scanned a 2Θ range of 10–32° and 44–60° with a step size of 0.05° and a counting time of 80 s per step.

Cross-sectional transmission electron micrographs were obtained using an image C${}_{s}$-corrected Titan 80–300 (FEI, Eindhoven, The Netherlands) microscope, which was equipped with a Gatan Imaging Filter 863. The primary electrons were accelerated to 300 kV. In addition to high-resolution TEM, energy-filtered TEM (EFTEM) with electrons exhibiting an energy loss of 17 eV due to the excitation of the valence band plasmons in Si was used for imaging the amorphous and crystalline silicon fractions. Complementarily, SiO${}_{2}$ was imaged using electrons with an energy loss of 27 eV (not shown here). EFTEM analysis was done with a slit width of 5 eV. TEM specimens of as-deposited and oven-treated samples were obtained by classical TEM specimen preparation, i.e., by sawing, grinding, dimpling and final Ar${}^{+}$ ion milling. For the laser-treated sample, a similar microscope (FEI Tecnai F30) was used for the analysis, and TEM lamella preparation was carried out using a focused ion beam with 30 keV for rough and 5 keV for subtle cutting. The zone of interest was protected by a sputtered 1.5 μm-thick layer of platinum.

## 5. Conclusions

Three-dimensional, percolated Si:SiO${}_{2}$ networks were obtained by reactive ion beam deposition of thin films with a measured stoichiometry of SiO${}_{0.64\pm 0.06}$. Subsequent oven and diode line-focused laser treatment led to different degrees of Si crystallization and structural coarsening. For the first time, diode line-laser scanning was applied as thermal treatment to SiO${}_{x}$ films for the creation of Si:SiO${}_{2}$ nanostructures.

In contrast to predictions and previous studies on percolated Si:SiO${}_{2}$ networks [7,8,18], phase separation into amorphous silicon and silica occurred during the deposition of SiO${}_{0.6}$ layers. During oven treatment, coarsening of the as-deposited structure morphology in a solid state regime occurred by diffusion of O atoms, as well as by grain boundary diffusion. Simultaneously, partial crystallization of the Si-phase by nucleation and growth into nano-sized, randomly-oriented crystallites were observed. In contrast, almost perfect, fully-crystalline Si and further coarsened structures were obtained by laser processing for considerably shorter treatment times. The larger structures were associated with a much higher temperature achieved during millisecond laser processing, eventually occurring in the liquid state of silicon and resulting in an order of magnitude higher diffusion constant. Perfect crystallinity resulted from the higher density of crystalline silicon, since the volume of the expanding silicon phase was restricted during solidification by the silica matrix. The vast extension of the obtained Si crystal grains resulted from crystallization along the liquid-solid interface during a process similar to explosive crystallization. In-plane tensile and out-of-plane compressive strain were explained by different thermal contractions, during cooling in the solid, of the fused silica substrate and the Si-phase inside the Si:SiO${}_{2}$ layer. The growth of interface layers was favored by the lower surface energy of the planar surface silica and fused silica substrate compared to the nanostructures of SiO${}_{2}$. The oxygen depletion and therefore silicon enrichment of the SiO${}_{0.6}$ layer in the vicinity of these silica interfaces led to the formation of the Si interlayer for laser-treated films.

The study demonstrates the high potential of diode line-laser scanning to form percolated Si:SiO${}_{2}$ nanocomposites. By using a line-focused diode laser beam, homogeneous and fast treatment of full samples is shown, to produce Si:SiO${}_{2}$ nanostructures with superior crystallinity. This proves the usability of this technology for general surface treatment and Si:SiO${}_{2}$ nanostructure formation.

On the other hand, different thermal treatments are required to cover the whole structure size scale of nano-silicon. The structure sizes achieved are not yet suitable for devices utilizing quantum confinement, e.g., solar cell absorbers with an increased energy gap. However, by knowing the different structure-forming regimes and relevant processes, this should be aimed at in future work. Despite this, the materials, as they are presented, could be candidates for utilization in supporting layers of solar cells or as electrical energy storage anodes.

## Figures and Tables

**Table 1:** Overview of RBS results of SiO${}_{0.6}$ layers on SiO${}_{2}$, as-deposited, oven- and laser-treated. Atomic fraction (at. %), areal density ($\rho_{area}$) of silicon and oxygen, the resulting x of ${\mathrm{SiO}}_{x}$ fraction and thickness obtained by TEM, $d_{TEM}$, as well as spectroscopic ellipsometry (SE), $d_{SE}$, for individual layers observed.

Sample	Si		O		x of SiO_x_	TEM Thickness	SE Thickness
	at. %	$\rho_{\mathrm{area}}/10^{15}\frac{1}{{\mathrm{cm}}^{2}}$	at. %	$\rho_{\mathrm{area}}/10^{15}\frac{1}{{\mathrm{cm}}^{2}}$		$d_{\mathrm{TEM}}/\mathrm{nm}$	$d_{\mathrm{SE}}/\mathrm{nm}$
**as-deposited**							
surface	33.3	33.3	66.6	66.5	2	14	15
bulk	61.0	1691	38.9	1079	0.64	500	509
**oven-treated**							
surface	33.3	50	66.6	99.8	2	39	40
bulk	60.5	1663	39.4	1084	0.65	501	482
**laser-treated**							
surface	33.3	50	66.6	99.8	2	28	29
intermediate 1	90.0	90	9.9	9.9	0.11	27	24
bulk	61.5	1648	38.4	1031	0.63	479	446
intermediate 2	90	45	10	5	0.11	24	10.7

**Table 2:** Treatment parameters and selected structure properties of as-deposited, oven- or laser-treated SiO${}_{0.6}$ layers. CVF, crystalline Si volume fraction.

	As-Deposited	Oven	Laser
**treatment realization**			
exposition time	-	270 min ≡ 16,200 s	$17\times 10^{-3}$ s
temperature	-	950${}^{\circ }C$	n/a
**resulting structure properties**			
bulk composition	SiO${}_{0.64}$	SiO${}_{0.65}$	SiO${}_{0.63}$
EFTEM structure size	2 nm	10 nm	30 nm
XRD grain size	n/a	11 nm	22 nm
Raman CVF	0%	72%	92%
